# Clinical presentation, outcome, and prognostic markers in patients with intravascular large B‐cell lymphoma, a lymphoma study association (LYSA) retrospective study

**DOI:** 10.1002/cam4.4742

**Published:** 2022-05-10

**Authors:** Antoine Bonnet, Céline Bossard, Ludovic Gabellier, Julien Rohmer, Othman Laghmari, Marie Parrens, Clémentine Sarkozy, Rémy Dulery, Virginie Roland, Francisco Llamas‐Gutierrez, Lucie Oberic, Luc‐Matthieu Fornecker, Laura Bounaix, Bruno Villemagne, Vanessa Szablewski, Sylvain Choquet, Krimo Bouabdallah, Alexandra Traverse‐Glehen, Mohamad Mohty, Laurence Sanhes, Roch Houot, Thomas Gastinne, Christophe Leux, Steven Le Gouill

**Affiliations:** ^1^ Service d’hématologie clinique, Centre Hospitalier Universitaire Nantes, Nantes, France (at the time of work) Service d’hématologie ‐ Centre Hospitalier Bretagne Atlantique, Vannes France (now); ^2^ Service d'anatomie et cytologie pathologique Centre Hospitalier Universitaire Nantes Nantes France; ^3^ Service d'hématologie Centre Hospitalier Universitaire Montpellier Montpellier France; ^4^ Service d'hématologie, Hôpital Pitié ‐ Salpêtrière – APHP Sorbonne Université Paris France; ^5^ Département de pathologie, Hôpital Haut‐Lévêque CHU et université de Bordeaux Bordeaux France; ^6^ Institut Gustave Roussy, Villejuif, France (at the time of work) Service d’hématologie Hospices Civils Lyon, Lyon France (now); ^7^ Service d’hématologie clinique et thérapie cellulaire Hôpital Saint‐Antoine, AP‐HP, Université Sorbonne, INSERM, Centre de recherche Saint‐Antoine Paris France; ^8^ Centre Hospitalier de Perpignan Service d'hématologie Perpignan France; ^9^ Centre Hospitalier Universitaire Rennes Service d'anatomopathologie Rennes France; ^10^ Service d'hématologie Centre Hospitalier Universitaire Toulouse, Institut Universitaire du Cancer de Toulouse Oncopole Toulouse France; ^11^ Service d'hématologie Institut de Cancérologie Strasbourg Europe (ICANS) Strasbourg France; ^12^ Service de thérapie cellulaire et d’hématologie clinique adulte Centre Hospitalier Universitaire Clermont‐Ferrand, site Estaing Clermont‐Ferrand France; ^13^ Service d'onco‐hématologie médecine interne Centre Hospitalier Départemental Vendée La Roche sur Yon France; ^14^ Service d'anatomopathologie Centre Hospitalier Universitaire Montpellier Montpellier France; ^15^ Service d’hématologie clinique et thérapie cellulaire Hôpital Haut‐Lévèque, CHU Bordeaux Bordeaux France; ^16^ Service d'anatomopathologie Hospices Civils Lyon Lyon France; ^17^ Service d’hématologie CHU Rennes, University of Rennes, INSERM U1236 Rennes France; ^18^ Service d’information médicale Centre Hospitalier Universitaire Nantes Nantes France; ^19^ INSERM CIRCNA nantes‐Angers NeXT Université de Nantes Nantes France; ^20^ Institut Curie Paris France; ^21^ Present address: Curie Institute Paris France; ^22^ Present address: Institut Gustave Roussy Villejuif France; ^23^ Present address: Curie Institute Paris France

**Keywords:** autoimmune disorders, BCL2 expression, intravascular lymphoma, nodal involvement

## Abstract

**Background:**

Intravascular large B‐cell lymphoma (lVLBCL) is a very rare type of large B‐cell lymphoma.

**Methods:**

We conducted a retrospective study on IVLBCL patients treated from 2000 to 2016 in LYSA cooperative group centers.

**Results:**

Sixty‐five patients were identified in 23 centers. Median age at diagnosis was 69 years (range 23–92). Thirty‐four patients (64%) had an IPI score >3 and 40 patients (67%) had a performance status ≥2. The most frequent extra‐nodal locations were bone marrow (*n* = 34; 52%), central nervous system (*n* = 25; 39%), and skin (*n* = 21; 33%). Nodal involvement and endocrine system were observed in 34% (*n* = 22) and 18% (*n* = 12) of all cases, respectively. Twenty‐six patients (41%) had macrophage activation syndrome. Tumor cells were frequently CD5 positive (52%) with a non‐germinal center origin (86%). BCL2 was expressed in 87% of all samples analyzed (*n* = 20) and 43% of patients had a MYC/BCL2 double expression. Fifty‐six patients were treated with a regimen of chemotherapy containing rituximab, among whom 73% reached complete remission. The median progression‐free survival (PFS) and median overall survival (OS) were 29.4 months and 63.8 months, respectively. History of autoimmune disorder (Hazard ratio [HR] 3.3 [1.4–7.8]; *p* < 0.01), nodal involvement (HR 2.6 [1.4–5.1]; *p* < 0.01), lack of anthracycline (HR 0.1 [0–0.4] for use; *p* < 0.001), or no intensification at first‐line regimen (*p* = 0.02) were associated with worse PFS. High‐dose methotrexate use was not associated with better PFS or OS.

**Conclusions:**

Our study highlights the aggressive clinical picture of IVLBCL, in particular the frequency of macrophage activation syndrome, and the need for new therapies despite a response to R‐CHOP‐like regimen similar to non‐intravascular diffuse large B‐cell lymphomas.

## Graphical abstract

Intravascular large B‐cell lymphomas in a large cohort present aggressive features and a dismal prognosis is suggested when associated with autoimmune disorders and nodal involvement.

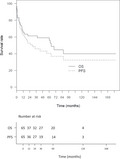

## 1 INTRODUCTION

Intravascular large B‐cell lymphoma (IVLBCL) is a rare non‐Hodgkin's lymphoma (NHL) entity characterized by the selective growth of neoplastic cells within blood vessel lumina, mainly capillaries with the exception of larger arteries and veins (WHO 4th edition 2017).^1^ The mechanisms responsible for tumor cell blood vessel infiltration remain unknown. It has been hypothesized that the intravascular growth pattern could be secondary to a defect of lymphoma cells homing receptors, such as lack of CD29 (B1 integrin), CD54 (ICAM‐1) adhesion beta‐molecules (Ponzoni, Hum Pathol 2000).^2^

IVLBCL is considered as a disseminated disease at diagnosis, with clinical and biological features that differ from one patient to another according to infiltrated organs and patients' geographic origin. Indeed, it has been reported that IVLBCL patients in western countries are more likely to have central nervous system (CNS) and cutaneous involvements as compared to patients from Asia. These latter would be conversely more exposed to hemophagocytic syndrome and medullary involvement (Ferreri, Haematologica 2007).^3^ The absence of marked lymphadenopathy, rarity of tumor cells, low tumor burden are sometimes responsible for a long time interval between onset of symptoms and IVLBCL diagnosis. This delay seems to be a significant factor in the prognostic of patients with IVLBCL (Geer, Br J Haematol 2019).^4^

Large IVLBCL cohorts are rarely found in the existing literature and consist mostly of case reports from Asian countries. These reports note a poor response rate to anthracycline‐containing regimen and a short median overall survival. (Ferreri, BJH 2008; Shimada, J Clin Oncol 2008).^5^, ^6^ In the present report, we aim to describe IVLBCL patients treated in LYSA centers from 2000 to 2016 and investigate biological prognostic markers that might be useful for customized treatments.

## 2 MATERIAL AND METHODS

### 2.1 Patient selection

All centers affiliated with the cooperative *Lymphoma Study Association (LYSA)* group were asked to report IVLBCL treated from 2000 to 2016. Local clinicians and pathologists were asked to report disease and patients' characteristics at diagnosis and to update the patients' outcome. Clinical data included age, sex, medical history of hematological malignancy, cancer or autoimmune disease, performance status, and involvement sites (according to clinicians, based on clinical, morphological, and histological data), Ann‐Arbor stage, presence or absence of B‐symptoms, and International Prognosis Index (IPI). Biological data including blood cells count, renal and hepatic function, serum lactate dehydrogenase (LDH), presence of hemophagocytosis, cerebrospinal fluid (CSF) analysis, HIV, HBV, and HCV serology, was recorded. First‐line treatment strategy and adverse events (AE) were systematically reported. Responses were assessed in accordance with the international workshop criteria (Cheson, 2007).^7^

### 2.2 Pathological study

IVLBCL were defined by at least one biopsy whose histological analysis reveals a predominant intravascular pattern of lymphoma cells, according to the WHO classification and the International Consensus Meeting on IVLBCL in 2007 (Ponzoni, J Clin Oncol 2007).^8^ No systematic centrally reviewed biopsy was performed for the purpose of the study, but cases were diagnosed in expert lymphoma centers and reviewed by the national *Lymphopath* network (for IVLBCL diagnosed after 2009). *Lymphopath* is a compulsory French National Cancer Agency program that imposes a pathologic reviewing by experts of all newly diagnosed lymphoma cases in France, before starting the treatment. In addition, pathologists centrally reviewed samples from 27 patients, confirmed diagnosis and performed complementary immunohistochemistry (IHC) analyses if necessary. Formalin‐fixed and Paraffin‐embedded samples were analyzed using the following antibodies (significant cutoff used in parentheses): CD20; CD5; BCL1; CD10 (30%); EBER; LMP1; BCL6 (30%); BCL2 (50%); MUM1 (30%); MYC (40%); CD29 (5%); ICAM1 (5%). Germinal center origin was determined according to the immunohistological algorithm proposed by Hans et al, and based on expression of CD10, BCL‐6, and MUM1 (Hans, Blood 2004).^9^ Centralized analysis have been performed on a control group of 31 biopsies of diffuse large B‐cell lymphomas (DLBCL) “not otherwise specified” (NOS), reviewed in the Lymphopath network. This sample has been selected for a similar rate of non‐germinal subtype and a large predominance of stage III‐IV of Ann Arbor classification.

### 2.3 Statistical analysis

Progression‐free survival (PFS) was calculated from the date of diagnosis to the date of death or disease progression. Overall survival (OS) was defined as the time from diagnosis until the date of death, regardless of the cause of death. Median follow‐up was calculated with reverse Kaplan–Meier method. Survival curves were generated using the Kaplan–Meier method and were compared by the log‐rank test. Multivariate analysis was carried out using Cox regression methods. Statistical analyses were carried out with a two‐tailed test at the 0.05 level, using R software version 3.3.3 (R foundation for statistical computing). This retrospective noninterventional study was reported to the *Direction de la recherche Clinique (DRCI) in* Nantes (*n*° RC16‐0034), according to the French legislation (article L 0.1121–0.1 and R1121‐2 of *Code de Santé Publique*).

## 3 RESULTS

### 3.1 Clinical features

Sixty‐five patients from 23 LYSA centers were diagnosed with IVLBCL between 2000 and 2016. Patients' characteristics and initial disease presentation are summarized in Table 1. Sex ratio male/female was 1.17 and median age at diagnosis was 69 years (range: 23–92). A previous medical history of hematological malignancy was found in eight patients (12%) and solid cancer in 13 cases (20%). Seven patients (11%) had an underlying autoimmune disorder (systemic lupus erythematosus; granulomatosis with polyangiitis; Hashimoto disease; Sjogren's syndrome; Schönlein‐Henoch purpura nephritis; celiac disease; rheumatoid arthritis).

**TABLE 1:** Patients' characteristics and initial disease presenting (*n* = 65)

	*n*	%^a^
Sex		
Female	30	46
Male	35	54
Performance status (ECOG)		
0–1	20/60	33
≥2	40/60	67
B‐symptoms	44/64	69
Primary involved site		
Bone marrow and/or spleen	34	52
CNS	25	39
Lymph nodes	22	34
Skin	21	33
Liver	17	27
Adrenal gland	10	16
Lung	7	11
Digestive tract	7	11
Kidney	6	9
Bone	5	8
Others^b^	9	14
Macrophage activation syndrome	26/64	41
Circulating tumor cells	11/60	18
CSF involvement	2/46	4
IPI score		
Low/intermediate (0–3)	19/53	36
High (4, 5)	34/53	64

Abbreviations: CNS, central nervous system; CSF, cerebrospinal fluid; IPI, International Prognostic Index; and NA, not available.

^a^ Results are reported on the total number of patients for each category, except when data were available only for a subset of patients which number is specified after slash.

^b^ Others: prostate, pituitary gland, bladder, ovaries, testicle, uterus, cavum, muscles, and heart.

At the time of diagnosis, performance status (PS) was ≥2 in 40 patients (67%), B‐symptoms were found in 44 cases (69%). All patients were in stage IV according to the Ann Arbor classification. Bone marrow was the most frequent involved site (52%). Central nervous system (CNS) and skin were invaded in 39% and 33% of cases, respectively. Endocrine system was involved in 18% of cases (adrenal in 10 patients, pituitary gland in two patients). Cytopenias were present with anemia in 53% (<100 g/L) and thrombocytopenia in 35% (<100 x 10^9^/L), associated with circulating tumor cells in 18% of patients.

### 3.2 Pathological features

IVLBCL diagnosis has been established on extra‐nodal site biopsies in every cases. It was mostly performed on skin or bone marrow biopsies (*n* = 36). Immunophenotypical characteristics are reported in Table 2. All samples tested for CD20 expression were positive, and half‐expressed CD5 (52%) without co‐expression of BCL1. BCL2 was overexpressed in 83% in the entire cohort. Cell of origin subtyping according to Hans algorithm resulted in a non‐germinal center type for the majority of cases (86%), due to the rarity of CD10 expression (11%). Epstein–Barr virus testing did not identify EBV+ tumor cells. For the 27 patients whose tissue samples were centralized, additional immunohistochemistry was performed in 23 of them with available tumor tissue. MYC was overexpressed in immunohistochemistry in 57% of cases, whereas BCL2 was overexpressed in 87% of them. MYC/BCL2 double expression status concerned 43% of cases.

**TABLE 2:** Immunohistochemical features

	*n*/*n* ^a^	%
Entire cohort		
CD20	60/60	100
CD5	14/27	52
EBER/LMP	0/14	0
CD10	3/28	11
BCL‐6	11/23	48
MUM1	14/22	64
GC (Hans)	3/22	14
BCL2	20/24	83
MYC	4/6	67
Centralized samples		
BCL2	20/23	87
MYC	12/21	57
Double‐expressor MYC/BCL2	9/21	43

^a^ Results are reported on available data which number is specified after slash.

**FIGURE 1:**
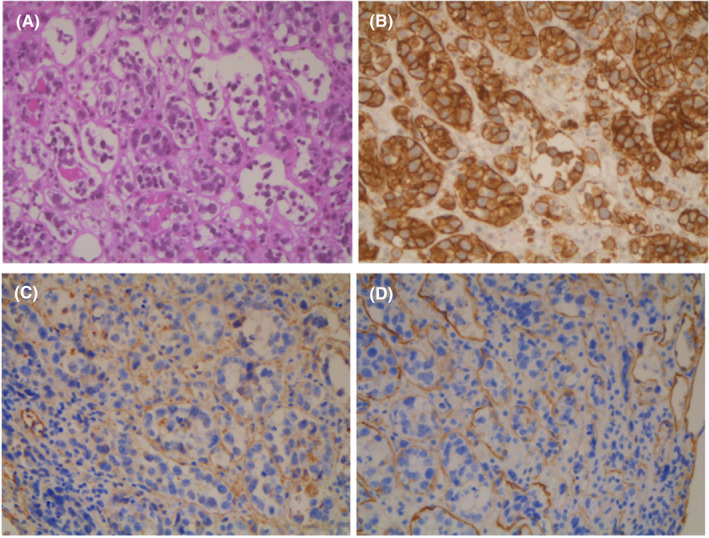
Adrenal gland involvement by intravascular large B‐cell lymphoma. (A) HES stain (hematoxylin eosin saffron). (B) CD20 expression of lymphoma cells. (C) CD29 is expressed on endothelial cells, but not on lymphoma cells. (D) CD54 is expressed on endothelial cells, but not on lymphoma cells

Tumor cells expression of adherence molecules (CD29 and ICAM1) was also tested on the centralized cohort, along with samples of 31 biopsies from diffuse large B‐cell lymphomas (DLBCL) “not otherwise specified” (NOS) as controls. In each tissue sample, endothelial cells served as positive internal control. CD29 was expressed in 33% in IVLBCL (six cases out of 18) compared with 62% in DLBCL NOS; ICAM1 was found in one IVLBCL sample out of 19 tested (5%) compared with 74% in DLBCL NOS (Figure 1).

### 3.3 Treatment

Two of the cases were only confirmed postmortem and four patients received supportive care. Regarding first‐line treatment, 59 received antineoplastic treatment and 56 were treated with an association of chemotherapy plus anti‐CD20 antibody (rituximab). One patient diagnosed in 2001 received chemotherapy without rituximab, one patient was treated with single agent rituximab (81‐year‐old with PS = 4) and data are missing for one case. Forty‐eight patients received an anthracycline‐based regimen. Other regimens were based on cytarabine and platinum association (*n* = 4), they were planned in a CNS lymphoma treatment schema for three patients (high‐dose methotrexate and/or cytarabine) and one patient received a Burkitt lymphoma chemotherapy regimen. CNS treatment or prophylaxis was given to 33 patients, with intrathecal infusion of chemotherapy (methotrexate and/or cytarabine) for 24 of them, and 18 patients (30%) received high‐dose methotrexate at first line treatment. Seven patients underwent autologous stem‐cell transplantation (ASCT) at frontline, after BEAM regimen for six of them. At relapse, ASCT were performed in seven patients (four BEAM, two Thiotepa/Busulfan/Cyclophosphamide, and one BCNU/Melphalan) and allogenic stem‐cell transplantation in two patients.

### 3.4 Clinical outcome

Fifty‐three patients were assessable for the response after first‐line treatment (one follow‐up loss and five premature deaths; for example, infection, gut perforation, and acute respiratory distress syndrome with multiorgan failure). Complete response was achieved in 43 patients (73%). Four patients had a stable disease and six patients had a progressive disease. All patients reached CR after ASCT.

**FIGURE 2:**
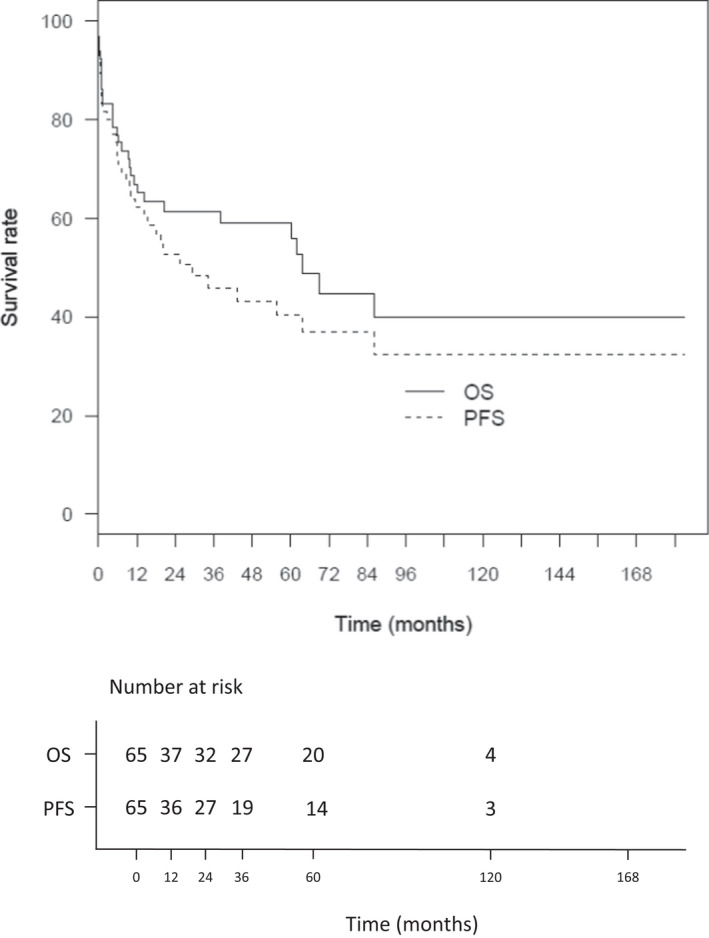
Overall survival (OS) and Progression‐free survival (PFS) of entire cohort. (*n* = 65)

**FIGURE 3:**
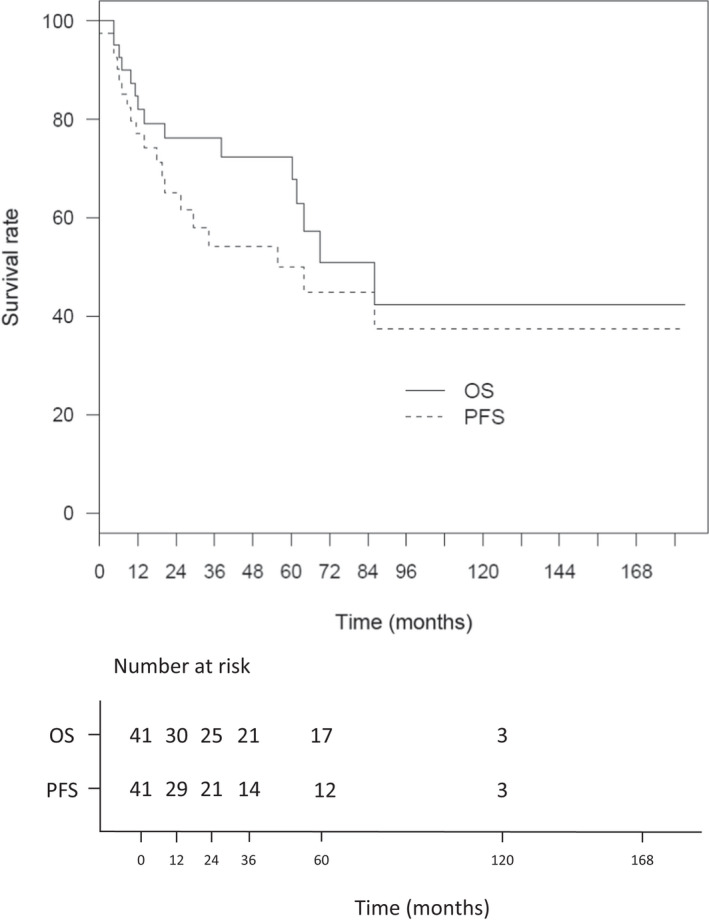
Overall survival (OS) and Progression‐free survival (PFS) of patients treated with RCHOP‐based regimen. (*n* = 43)

With a median follow‐up was 57.7 months (IQR 38.4–76.3),the median PFS was 29.4 months after diagnosis, with a 2‐year and a 5‐year PFS rate at 52.7% (95% CI: 41.4–67.1) and 40.3% (95% CI: 28.7–56.6), respectively. The median OS was 63.8 months, with a 2‐year and a 5‐year OS rate at 61.4% (95% CI: 50.3–74.9) and 59.0% (95% CI: 47.7–73.1), respectively (Figure 2). In the cohort of patients treated with R‐CHOP‐like regimen (*n* = 43), the 2‐year and 5‐year PFS was 65.1% (95% CI: 51.3–82.6) and 50% (95% CI: 35.1–71.2). The 2‐year and 5‐year OS was 76.1% (95% CI: 63.6–91.2) and 72.3% (95% CI: 58.9–88.9) (Figure 3).

### 3.5 Prognostic factors

All the prognostic factors tested on PFS and OS are listed in Table 3.

**TABLE 3:** Prognostic factors for PFS and OS

	Progression‐free survival						Overall survival					
	Univariate			Multivariate			Univariate			Multivariate		
Variable	HR	95% CI	*p*‐value	HR	95% CI	*p*‐value	HR	95% CI	*p*‐value	HR	95% CI	*p*‐value
Sex, male	0.8	[0.4–1.5]	0.490				1	[0.5–2.2]	0.919			
Age ≥ 70 years	1.3	[0.7–2.4]	0.496	0.6	[0.2–1.4]	0.227	1.8	[0.9–3.7]	0.117	0.9	[0.4–2.3]	0.864
Autoimmune disorder	**3.3**	**[1.4–7.8]**	**<0.01**				**2.9**	**[1.1–7.1]**	**0.024**			
Cancer associated	0.8	[0.3–1.8]	0.562				1	[0.4–2.3]	0.986			
Other HM associated	1.5	[0.6–3.9]	0.404				0.9	[0.3–2.8]	0.802			
Performance status ≥2	2.1	[0.9–4.7]	0.079	1.6	[0.6–4.2]	0.386	**2.6**	**[1–6.6]**	**0.048**	2.2	[0.8–6.6]	0.148
B‐symptoms	1	[0.5–2]	1.000				1	[0.5–2.2]	0.993			
Involvement site												
CNS	1	[0.5–2]	0.996				0.9	[0.4–1.8]	0.692			
Skin	1.3	[0.7–2.6]	0.432				1.6	[0.8–3.4]	0.209			
BM/Spleen	1.4	[0.7–2.7]	0.352				1.2	[0.6–2.5]	0.638			
Lung	0.6	[0.2–1.8]	0.331				0.5	[0.1–2.1]	0.357			
Kidney	1.1	[0.3–3.6]	0.89				1.4	[0.4–4.6]	0.604			
Lymph nodes	**2.6**	**[1.4–5.1]**	**<0.01**	**4.8**	**[1.9–12.3]**	**<0.001**	**2.9**	**[1.4–6]**	**<0.01**	**7.4**	**[2.5–22.1]**	**<0.001**
Bone	2.2	[0.8–6.5]	0.135				2.4	[0.8–7.1]	0.1			
Hemophagocytosis	1.3	[0.7–2.5]	0.456				1.1	[0.5–2.3]	0.768			
Tumor cells in PB	1	[0.4–2.2]	0.94				1	[0.4–2.5]	0.991			
Elevated LDH	1.3	[0.5–3.2]	0.528				1.3	[0.5–3.5]	0.548			
IPI 4–5	1.4	[0.6–3.1]	0.424				1.9	[0.8–5]	0.17			
Treatment^a^												
Anthracycline‐based regimen	**0.1**	**[0–0.4]**	**<0.001**	**0.06**	**[0.02–0.2]**	**<0.0001**	**0.1**	**[0–0.3]**	**<0.0001**	**0.03**	**[0.01–0.1]**	**<0.0001**
R‐CHOP regimen	0.6	[0.3–1.4]	0.248				0.5	[0.2–1.3]	0.157			
High‐dose Methotrexate	0.9	[0.4–2,2]	0.886				1.1	[0.4–2,6]	0.876			
ASCT at first‐line	—	—	**0.02**				—	—	0.054			
ASCT at any time	NA						**0.1**	**[0–0.6]**	**0.035**			

The bold values indicate statistically significant values.

Abbreviations: ASCT indicates autologous stem‐cell transplantation; CI, confidence interval; CNS, central nervous system; HM, hematological malignancies; HR, hazard ratio; IPI, international prognostic index; PB, peripheral blood.

^a^ Comparison among patients who received chemotherapy.

**FIGURE 4:**
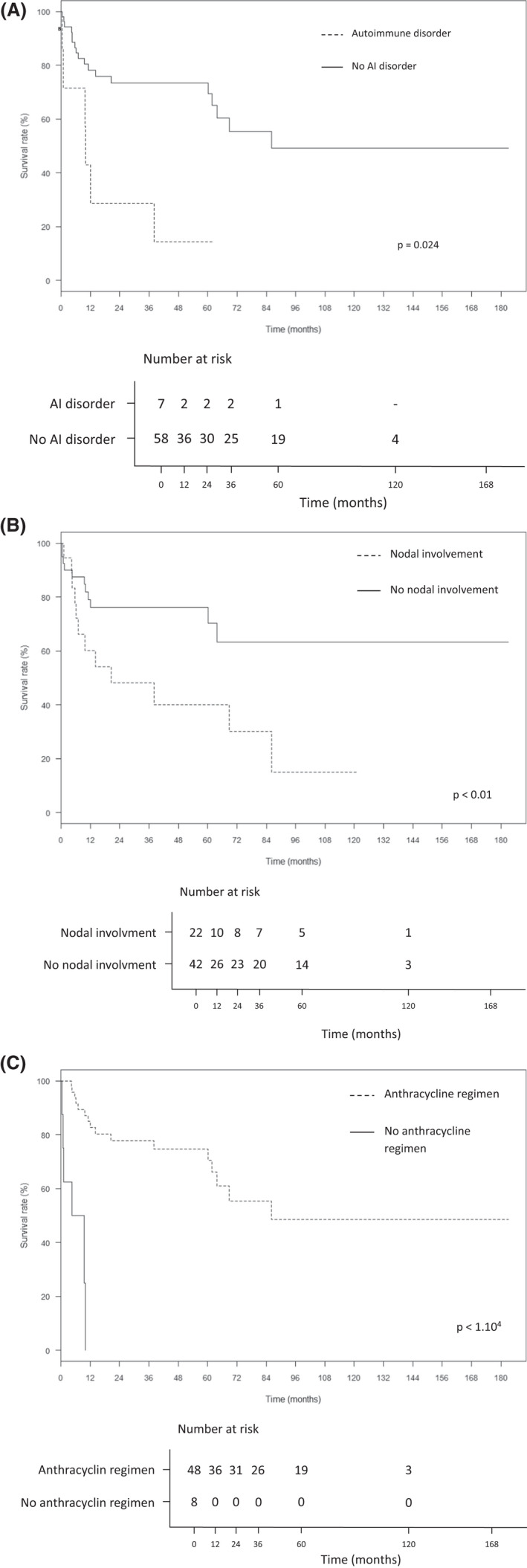
Medical history of autoimmune disorder (A), nodal involvement (B), and lack of anthracycline in chemotherapy regimen (C) are significantly associated with worse OS in patients who received chemotherapy (*n* = 57)

In univariate analysis, history of autoimmune disorder (hazard ratio [HR] 3.3 [1.4–7.8]; *p* = 0.006) (Figure 4A), nodal involvement (HR 2.6 [1.4–5.1]; *p* = 0.004) (Figure 4B), and anthracycline use at first‐line regimen (HR 0.1 [0–0.4]; *p* < 0.001) (Figure 4C) were significantly associated with PFS. Patients receiving ASCT had a better PFS (*p* < 0.02). High‐dose methotrexate use was not associated with better PFS.

In multivariate analysis, lymph nodes involvement was predictive of worse PFS (HR 4.8 [1.9–12.3]; *p* < 0.001) and OS (HR 7.4 [2.5–22.1]; *p* < 0.001), while anthracycline‐containing regimen improved PFS (HR 0.06 [0.02–0.2]; *p* < 0.0001) and OS (HR 0.03 [0.01–0.1]; *p* < 0.0001).

## 4 DISCUSSION

IVLBCL at diagnosis present with aggressive features such as advanced disease (stage IV in 100%), high IPI score (IPI 4–5 in 64%), presence of B‐symptoms (69%), and high incidence of both neurological (39%) and skin (33%) involvement. In our cohort, incidence of nodal involvement (34%) in IVLBCL is more frequently reported than in previous studies (Table 4).^10^, ^11^, ^12^ Interestingly, coexisting malignancies are frequent and autoimmune disorders are significantly associated with worst prognosis. Presence of hemophagocytosis with macrophage activation syndrome (41%) is also more frequent in our cohort than in Ferreri's (Table 4).^10^ According to WHO classification, IVLBCL is divided into two clinical pictures with different geographical distributions. The so‐called “classic form” is more frequent in Western countries and goes along with symptoms related to organ involvement. Contrarily, the so‐called “haemophagocytic syndrome‐associated form” is more frequently reported in Asian patients. In our cohort, which included only patients treated in France (no ethnic origin reported, according to French law), incidence of hemophagocytosis with macrophage activation syndrome is comparable to what has been reported in the Asian IVLBCL population.

**TABLE 4:** Clinical and biological features in main studies on IVLBCL

	Current study	Ferreri (2004)	Murase (2007)	Brunet (2017)
	*n* = 65	*n* = 38	*n* = 96	*n* = 29
Median age (years)	69	70	67	67
Men; *n* (%)	35 (54)	18 (47)	50 (52)	16 (55)
B‐symptoms; *n* (%)	44 (69)	21 (55)	73 (76)	29 (100)
Involved site				
Bone marrow; *n* (%)	34 (52)^a^	14 (37)	67 (75)	8 (27,6)
Nervous system; *n* (%)	25 (39)	15 (39)	26 (27)	22 (76)
Lymph nodes; *n* (%)	22 (34)	4 (11)	−(11)	7 (24)
Skin; *n* (%)	21 (33)	15 (39)	14 (15)	5 (17)
Endocrine system; *n* (%)	13 (20)	6 (16)	3 (3)	7 (24)
Circulating tumor cells; *n* (%)	11 (18)	2 (5)	23 (24)	—
Hemophagocytosis; *n* (%)	19 (36)	0 (0)	54 (61)	2 (7)
Stage IV; *n* (%)	64 (100)	−(76)	87 (91)^b^	29 (100)
IPI high (4, 5)	34 (64)	16 (42)	72 (75)	29 (100)

^a^ Bone marrow/spleen.

^b^ Stage III–IV.

CD5 positivity was found in 52% of cases but was not significantly associated with PFS (HR 1.9 [CI95% 0.6–6.1], *p* = 0.26), as previously reported by Murase et al.^11^ Cellular origin was mainly a non‐germinal center type (86%), and high expression level of MYC (57%), BCL2 (87%), or both MYC/BCL2 (43%) appears to be more frequent than in DLBCL NOS. Double‐expression MYC/BCL2 is known for its adverse prognostic impact, but also BCL2 expression (40%–60% of DLBCL NOS), independently of IPI score or MYC expression (Petrella, Ann Oncol 2017).^13^ High expression of MYC and BCL2 in IVLBCL could be in‐line with their aggressive presentation (high IPI, advanced disease, B‐symptoms). Interestingly, the lack of CD54 (ICAM‐1) and CD29 (integrin beta‐1) adherence molecules expression by tumor cells in our cohort compared with tumor cells of DLBCL NOS, suggests a loss of expression of those “homing” molecules and reinforces the hypothesis that this peculiar and exclusive intravascular growth pattern is secondary to this defect of homing receptors expression by neoplastic cells (Ponzoni, 2000).^2^ The scarcity of tumor cells in tumor biopsies did not enable the performance of molecular analysis, especially to investigate MYC, BCL2, or BCL6 rearrangement.

The 2‐year PFS (53%) and OS (61%) in our cohort are similar to Shimada's report (56% and 66%, respectively), illustrating “the poor prognosis” of IVLBCL as described in the WHO classification.^1^ However, according to their 2‐year PFS (65%) and OS (76%), patients treated with R‐CHOP‐like regimen have similar outcomes to patients with non‐IV DLBCL.^14^ This result is in‐line with a recent population‐based study in the US including 344 IVLBCL patients diagnosed between 2000 and 2013. This study, reported by Rajyaguru et al.,^15^ used a *Surveillance, Epidemiology, and End Results* program and *National Cancer Database*. The 3 and 5‐years OS were 52% and 46%, respectively and did not differ from those of the 133,993 patients with DLBCL NOS after using a propensity score which included variables such as age, clinical stage, or comorbidity index. A recent phase II trial conducted in Japan supports the use of R‐CHOP plus high‐dose methotrexate frontline and intrathecal chemotherapy, with a 2‐year PFS of 76% in a selected population (without CNS symptoms).^16^ But, in practice, high‐dose methotrexate in elderly or frail patients can be challenging, and treatment of IVLBCL is not well‐defined. Our work suggests that intensive therapy (like ASCT) could provide better outcome, but more patients are needed to confirm this. Indeed, other biology‐driven therapeutic approaches based on IVLBCL biology should be further investigated, such as BCL‐2 or microenvironment‐targeted therapies.

Our work confirms the aggressive features of IVLBCL and suggests a dismal prognosis when associated with autoimmune disorders at the time of diagnosis. R‐CHOP‐like regimen could be recommended, but better IVLBCL––tailored therapies are needed.

### AUTHOR CONTRIBUTION

AB performed acquisition, analysis, or interpretation of data for the study. AB and SLG contributed the conception and design of the work, drafted the manuscript; CB, LG, JR, OL, MP, CS, RD, VR, FLG, LO, LMF, LB, BV, VS, SC, KB, ATG, MM, LS, RH, and TG contributed to collect data. OL and CB provided pictures for Figure 1. CL performed statistical analysis.

### CONFLICT OF INTEREST

No conflict of interest for all authors.

### ETHICS STATEMENT

This retrospective noninterventional study was reported to the Direction de la recherche Clinique (DRCI) in Nantes (n° RC16‐0034), according to the French legislation (article L 0.1121–0.1 and R1121‐2 of Code de Santé Publique).

## Data Availability

The data that support the findings of this study are available from the corresponding author upon reasonable request.
